# Assessing the accuracy and reliability of application-based audiometry for hearing evaluation

**DOI:** 10.1038/s41598-024-57944-9

**Published:** 2024-03-28

**Authors:** Seung Yeol Lee, Hee Won Seo, Seon Min Jung, Seung Hwan Lee, Jae Ho Chung

**Affiliations:** Department of Otolaryngology-Head and Neck Surgery, School of Medicine, College of Medicine, Hanyang University, 222-Wangshimni-ro, Seongdong-gu, Seoul, 133-792 Korea

**Keywords:** Diseases, Health care, Medical research

## Abstract

Pure-tone audiometry (PTA) is the gold standard for assessing hearing loss. However, traditional PTA tests require specialized equipment, trained personnel, and a soundproof environment. Recently, smartphone-based PTA tests have been developed as an alternative method for hearing assessment. The aim of this study was to validate the accuracy and reliability of a smartphone application-based audiometry test. This study was conducted to assess the performance of application-based audiometry from November 2021 to January 2022. Pure-tone thresholds were measured using a smartphone application-based PTA test and compared with results obtained using a traditional audiometer in a sound-treated booth. The smartphone application used in this study was the "Care4Ear (Care4ear, version 1.0.6, MIJ Co., Ltd.)". Hearing thresholds less than 35 dB HL were classified as group A, 35-64 dB HL as group B, and 65 dB HL or greater as group C for the classification of hearing levels. We evaluated the accuracy of smartphone audiometry for each group and compared the results of frequency-specific hearing tests. Additionally, we examined the results of smartphone audiometry in individuals (n = 27) with asymmetric hearing loss. Seventy subjects completed both conventional audiometry and smartphone application-based hearing tests. Among the ears assessed, 55.7% were classified as group A, while 25.7% and 18.6% were classified as group B and group C, respectively. The average hearing threshold obtained from conventional pure-tone audiometry was 37.7 ± 25.2 dB HL, whereas the application-based hearing test yielded thresholds of 21.0 ± 23.0 dB HL. A significant correlation (r = 0.69, *p* < 0.01) was found between the average hearing thresholds obtained from the application-based and conventional pure-tone audiometry tests. The application-based test achieved a 97.4% hit rate for classifying hearing thresholds as class A, but lower rates of 22.2% for class B and 38.5% for class C. Notably, a discrepancy was observed between the hearing threshold measured by the application and the conventional audiometry for the worse ear with asymmetric hearing. The smartphone-based audiometry is a feasible method for hearing evaluation especially in persons with normal hearing. In cases of hearing loss or asymmetric hearing loss, the results of the application-based audiometry may be inaccurate, limiting its diagnostic utility.

## Introduction

Hearing loss is a common sensory impairment affecting individuals of all ages. According to the World Health Organization (WHO), over 5% of the world's population, or approximately 466 million people, have disabling hearing loss, with most cases occurring in low- and middle-income countries^1^.

The changing demographic structure, particularly the increasing elderly population, is expected to lead to a higher prevalence of age-related hearing loss. Moreover, the usage of personal audio devices among younger generations is on the rise, and increased exposure to noise through various recreational activities is contributing to an increase in noise-induced hearing loss. Consequently, it is anticipated that hearing impairment will progressively become more prevalent across all age groups^2,3^.

The impact of hearing loss extends beyond communication difficulties and can significantly affect social interactions and emotional well-being. Individuals with untreated hearing loss often experience social isolation, depression, and decreased overall satisfaction with life^4^. In addition, hearing loss can hinder educational and occupational performance, limiting opportunities for personal and professional growth^5,6^. Recent studies have demonstrated a significant correlation between hearing loss and cognitive decline including dementia, emphasizing the need for greater awareness and attention to hearing loss in the context of cognitive aging^7,8^.

Given the widespread prevalence of hearing loss and its far-reaching consequences, early detection and intervention are crucial. However, barriers such as cost, limited access to healthcare facilities, and stigma surrounding hearing loss often prevent individuals from seeking timely diagnosis and treatment.

To diagnose hearing loss, conventional pure-tone audiometry tests are the gold standard for hearing assessment; however, they require specialized equipment and trained professionals, making them expensive and inaccessible in many settings.

With the proliferation of smartphones and the introduction of the concept of digital medicine, the versatile functions of smartphones have found application within the realm of healthcare. They are being utilized across various domains, contributing significantly to the enhancement of healthcare accessibility^9,10^. In this context, smartphone-application based hearing tests have emerged as a potential alternative for hearing assessment due to their convenience, portability, and accessibility^11–15^.

The aim of the present study was to assess the feasibility of the application-based audiometry in comparison with conventional pure tone audiometry tests.

## Methods

### Participants

The present study was conducted at the Department of Otolaryngology at the tertiary referral center between November 2021 and January 2022. The study enrolled patients who consented to undergo application-based audiometry in addition to conventional hearing tests. Prior to the audiometric evaluations, all participants underwent otoscopy to assess the condition of the tympanic membrane, and any excess earwax or foreign bodies in the ear canal were removed. The study targeted adult patients (age ≥ 18) who were able to cooperate with both pure tone audiometry and application-based audiometry. Individuals with limitations in smartphone usage, cognitive impairments, or neurological conditions such as Alzheimer's disease or Parkinson's disease were excluded from the study.

### Audiometric evaluation: conventional puretone audiometry

Patients underwent conventional pure tone audiometry (PTA) using the GSI AudioStar Pro™ (Grason-Stadler, Smørum, Denmark) with TDH39 headphones (Telephonics; Farmingdale, NY, USA). The audiometric testing was conducted within a sound-treated booth, and the frequency range of 250–8000 Hz was evaluated. This study used a modification of the Hughson-Westlake method, which is called the 'up-5 down-10 technique.' The pure tone sound level was initially introduced at a higher level of estimated hearing threshold and decreased by 10 dB HL after a correct response from the patient. Otherwise, the sound level was increased by 5 dB HL, if the patient gave an incorrect or no response^16^. In cases where the thresholds between ears differed by 40 dB HL or more in air conduction, contralateral masking was implemented to adjust the cross-hearing.

### Application based audiometry

All patients underwent mobile-based audiometry using the Care 4 Ear application (version 1.0.6, MIJ Co., Ltd.). This application was freely available for download on the Apple Store for iOS devices and on Google Play for Android devices. For the mobile-based audiometry in the present study, the hearing tests were conducted using an iPad mini 3 (iOS 8; Apple, Cupertino, CA, USA)) and EarPods with a 3.5 mm Headphone Plug (Apple, Cupertino, CA, USA). The hearing tests were conducted in a quiet office, and all participants underwent the app-based hearing test in the same environment. The application measured the external sound levels, and the hearing test proceeded only when the noise level was below 40 dB. The application provided an instruction for conducting hearing tests, the hearing tests were performed according to the app's algorithm at the frequency of 250 Hz, 500 Hz, 1000 Hz, 2000 Hz, 4000 Hz, and 8000 Hz. To address usability issues, a clinical research coordinator assisted in guiding through the examination process. During the mobile-based audiometry, subjects were able to easily respond to each sound stimulus by pressing a button on the touch screen of the iPad. The audiometric testing started with a 1000 Hz tone presented at 40 dB, and the sound pressure level was progressively increased by 10 dB until a response was observed. In cases where there was no response, the sound level was reduced by 5 dB to determine the hearing threshold. The application-based audiometry test takes about 10 min to examine both ears. The thresholds obtained from the mobile-based tests were recorded and could be calculated to determine the average thresholds using the same methodology as conventional PTA.

### Assessment the performance of application-based audiometry test

To calculate the average hearing threshold, both conventional and application-based audiometry utilized the following formula: (500 Hz + 2 × 1000 Hz + 2 × 2000 Hz + 4000 Hz) / 6. Based on the results of conventional pure-tone audiometry (PTA), the hearing levels were categorized according to the WHO criteria as follows: individuals with a PTA of less than 35 dB HL were classified as Group A (normal hearing and mild hearing loss), those with a hearing threshold surpassing 35 dB HL but less than 65 dB HL were categorized as Group B (moderate and moderately severe hearing loss), and individuals with a threshold greater than 65 dB HL were classified as Group C (severe and profound hearing loss)^1^. The concordance between hearing thresholds measured using conventional PTA and application-based audiometry was assessed based on the each hearing group, and this concordance was defined as the "hit rate."

### Subgroup analysis

Asymmetric hearing loss was defined as more than 20 dB HL average threshold difference, which was calculated using the following formula: (500 Hz + 2 × 1000 Hz + 2 × 2000 Hz + 4000 Hz) / 6, between the better and worse ears in conventional pure-tone audiometry, while the others were defined as symmetric hearing. The hearing thresholds at each frequency were compared between conventional PTA and application-based audiometry in both the better and worse ears in asymmetric hearing.

### Statistical analysis

Statistical analyses were performed with Statistical Package for the Social Sciences (SPSS) for Windows 26.0 (IBM Corp., Armonk, NY, USA. The Pearson correlation coefficient was utilized for bivariate correlation analysis to examine the correlation between the hearing thresholds measured by conventional pure-tone audiometry and the application-based audiometry. The correlations between the two groups were analyzed not only for the overall results but also for each frequency. The Paired Student’s t-test was applied to compare the individual average hearing thresholds and the individual hearing thresholds at each frequency between the two groups. Categorical variables were expressed as frequencies and percentages. A *p*-value < 0.05 was considered statistically significant.

### Ethics

Written informed consent was obtained from all patients, and the investigation was approved by the Institutional Review Board (IRB) of Hanyang University Guri Hospital and performed in accordance with the Declaration of Helsinki and Good Clinical Practice guidelines (IRB FILE No: 2021-11-021-001).

## Results

### Demographics

A total of 70 patients underwent both conventional audiometry and app-based audiometry for hearing assessment. Among them, 34 were male (48.6%), and 36 were female (51.4%). The average age of the patients was 54.3 ± 15.0 years. The patients visited the otolaryngology department with diverse ear conditions, including tinnitus in 18 cases (25.7%), bilateral sensorineural hearing loss in 25 cases (35.7%), unilateral sudden sensorineural hearing loss in 10 cases (14.3%), chronic otitis media in 15 cases (21.4%), and conductive hearing loss in 2 cases (2.9%). Among the patients, 23 (32.9%) had hypertension, and 9 (12.9%) had diabetes mellitus (Table 1).

**Table 1:** Demographic of the study population.

Variables	Number of patients (N = 70)
Gender	
Male / Female	34 (48.6%) / 36 (51.4%)
Age, years	
Mean ± Standard Deviation / Range	54.3 ± 15.0 (18 – 77)
Diagnosis	
Tinnitus	18 (25.7%)
Sensorineural hearing loss	25 (35.7%)
Unilateral sudden sensorineural hearing loss	10 (14.3%)
Chronic otitis media	15 (21.4%)
Unilateral conductive hearing loss	2 (2.9%)
Systemic Disease	
Hypertension	23 (32.9%)
Diabetes mellitus	9 (12.9%)
Hearing Threshold (Conventional PTA, dB)	
Mean Threshold ± Standard Deviation	37.7 ± 25.2 dB
Hearing Grades*, ears, n	140 (100%)
Class A < 35 dB HL	78 (55.7%)
Class B 35–64 dB HL	36 (25.7%)
Class C ≥ 65 dB HL	26 (18.6%)

*Four frequency average (0.5, 1, 2, and 4k) according to classification criteria of WHO,

Class A: normal hearing + mild hearing loss.

Class B; Moderate and moderate to severe hearing loss.

Class C; Severe and profound hearing loss.

### Correlation between the conventional and application-based audiometry.

**Figure 1:**
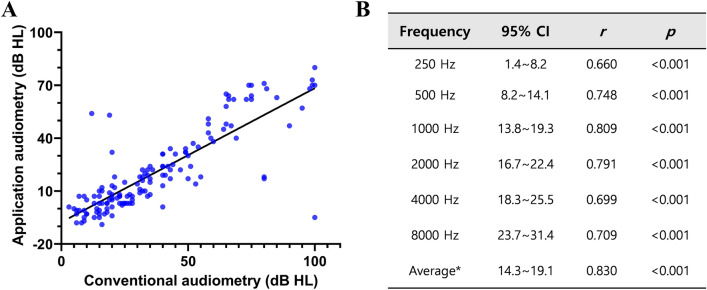
Correlation between conventional hearing tests and application-based audiometry. (**A**). Scatter plot for the average thresholds of conventional and application-based audiometry. (**B**). Correlation of both audiometric tests at each pure tone frequency. 95%CI: 95% confidence interval difference, r: Pearson correlation coefficient, p: p value for correlations. *Four frequency average (0.5, 1, 2, and 4 k).

**Figure 2:**
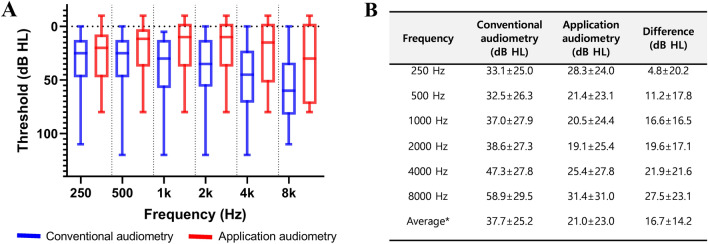
Hearing Threshold results of conventional and application-based audiometry. (**A**). Comparison of hearing threshold results. (**B**) The difference in hearing thresholds by frequency and average thresholds between conventional and application-based audiometry.

Pearson correlation analysis showed that the results from the two audiometric methods correlated for the average thresholds at frequencies of 0.5 kHz, 1 kHz, 2 kHz, and 4 kHz, with a coefficient (r) of 0.830 (Fig. 1). At each frequency, the two audiometry results showed correlations with coefficients (r) of 0.660 at 250 Hz, 0.748 at 500 Hz, 0.809 at 1 kHz, 0.791 at 2 kHz, 0.699 at 4 kHz, and 0.709 at 8 kHz. When comparing the mean thresholds at each frequency, it became evident that the higher the frequency, the greater the difference between PTA and the application (Fig. 2). The difference of the average threshold was 16.7 ± 14.2 (95% CI 14.3–19.1) dB HL and those of threshold at 250 Hz and 8 kHz were 4.8 ± 20.2 (95%CI 1.4–8.2) and 27.5 ± 23.1 (95% CI 23.7–31.4) respectively.

### Accuracy of application-based audiometry according to hearing level

**Figure 3:**
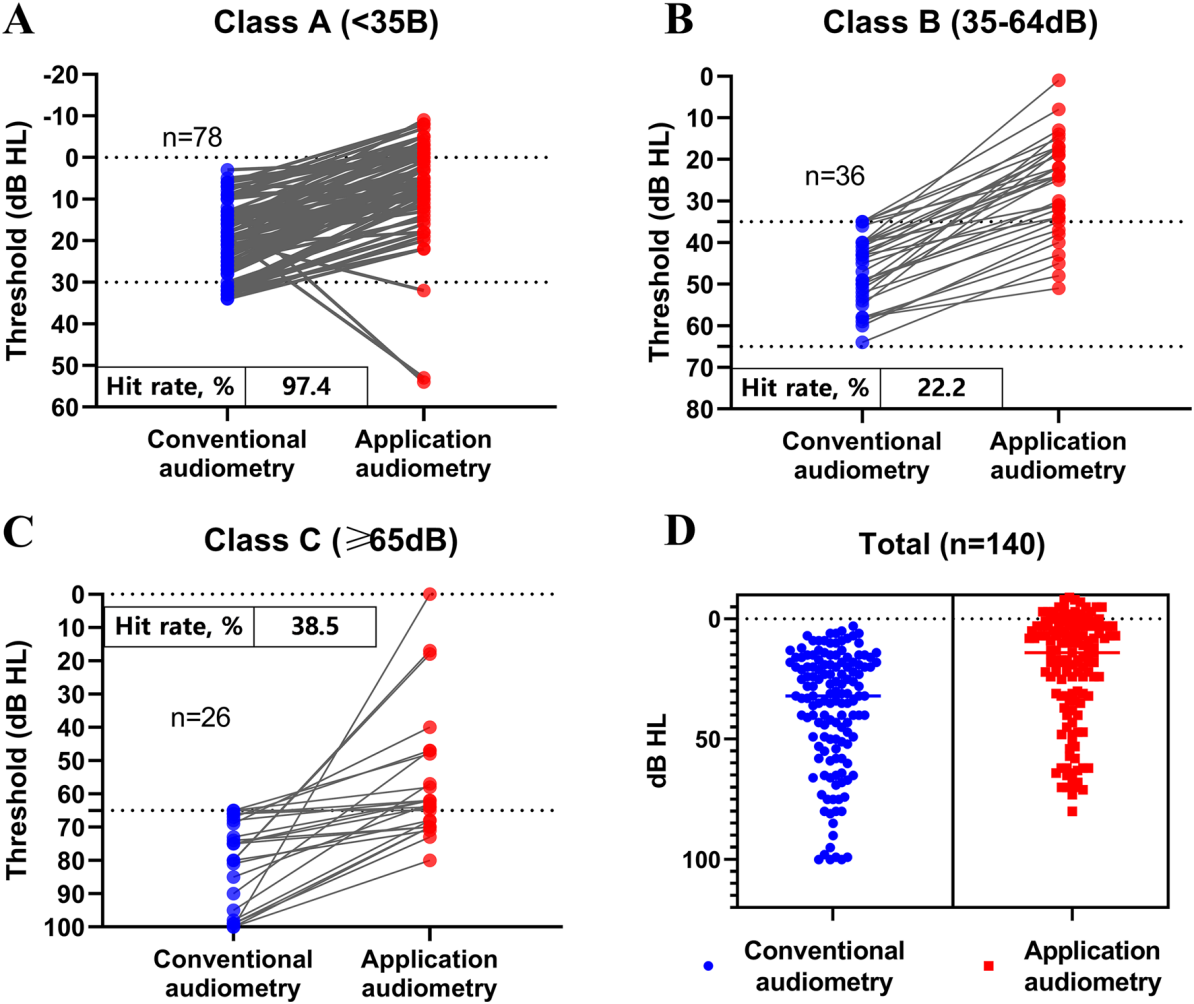
The hit rate of application-based audiometry test according to hearing level. (**A**). Class A (Normal and mild hearing loss, 97.4%). (**B**). Class B (Moderate and moderate to severe hearing loss, 22.2%). C: Class C (Severe and profound hearing loss, 38.5%). D: Overall nested plots.

Figure 3 illustrates the contrast between application-based audiometry and conventional audiometry across different hearing classes, with the accuracy of the hearing test results achieved through the application being defined as the 'hit rate”. The hit rate of the application-based test for correctly classifying as class A was 97.4%, while those for class B and C were 22.2% and 38.5%, respectively (Fig. 3). In the nested scatter plot, the total distribution of each audiometric result from the two audiometric methods was drawn, and the scattered plot shapes from the two results showed similar distribution (Fig. 3).

### Subgroup analysis for asymmetric and symmetric hearing

**Figure 4:**
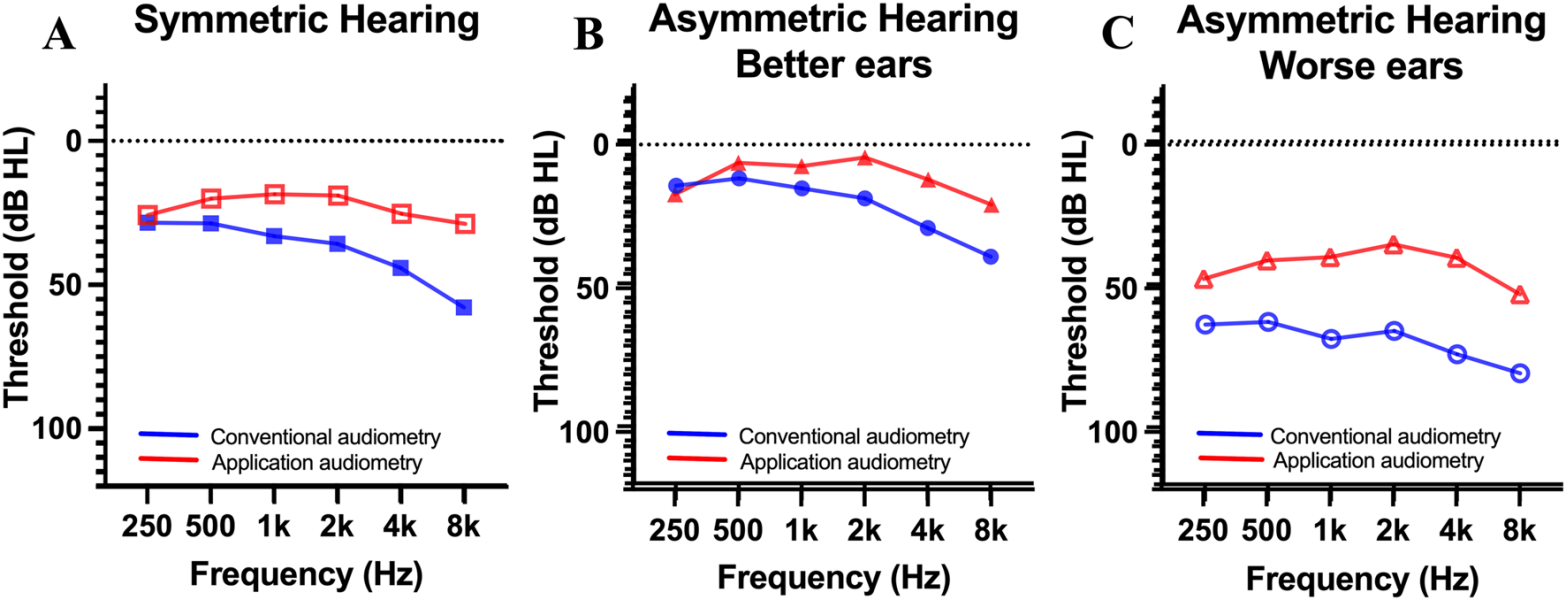
Hearing test results with symmetric and asymmetric hearing. (**A**). Hearing threshold for subjects with symmetric hearing. A. Hearing threshold for better ears in subjects with asymmetric hearing. (**B**). Hearing threshold for worse ears in subjects with asymmetric hearing.

Twenty-seven patients (54 ears) with asymmetric hearing thresholds (≥ 20 dB HL) between bilateral ears and other forty-three patients (86 ears) with symmetric hearing were analyzed (Table 2). In symmetric hearing group, threshold difference between application based and conventional audiometry was 14.8 ± 11.8 dB HL, while asymmetric hearing subjects showed the hearing threshold difference between application-based and conventional audiometry, 12.0 ± 8.3 dB HL, in the better hearing ear and 27.7 ± 19.9 dB HL in the worse hearing ear (Fig. 4).

**Table 2:** Hearing test results of symmetric and asymmetric hearing.

Variables	Values
Number of individuals with asymmetric hearing loss	27
Better Ears	
Pure tone audiometry, dB HL	19.2 ± 11.2
Application audiometry, dB HL	7.2 ± 9.9
Difference	12.0 ± 8.3
Worse Ears	
Pure tone audiometry, dB HL	65.9 ± 23.8
Application audiometry, dB HL	37.9 ± 24.9
Difference	27.7 ± 19.9
Number of individuals with symmetric hearing	43
Pure tone audiometry, dB HL	34.8 ± 21.4
Application audiometry, dB HL	20.0 ± 22.2
Difference	14.8 ± 11.8

Asymmetric hearing loss: the pure tone audiometry shows a difference of more than 20 dB between the two ears.

## Discussion

The present study sought to assess the viability of smartphone-based audiometry as a tool for hearing assessment across a diverse patient population. Our findings offer valuable insights into the application's potential, its alignment with conventional audiometry and its limitations. The present study can be summarized as follows: (1) Strong positive correlations were found between smartphone-based audiometry and conventional pure-tone audiometry (PTA) for most frequencies, indicating its reliability in estimating hearing thresholds. (2) The application demonstrated high accuracy in classifying Class A hearing levels, suggesting its effectiveness in identifying normal hearing to mild hearing loss. (3) Limitations exist when assessing the high-frequency range and categorizing more severe cases of hearing loss, emphasizing the necessity for additional refinement and validation of the application.

Smartphone-based medical applications have revolutionized healthcare delivery, offering a wide range of services from remote patient monitoring to diagnostic tools^10,17,18^. These applications empower individuals to track their health, access medical advice, and even perform self-assessments, such as measuring vital signs or body movement. The convenience and accessibility provided by smartphone-based healthcare solutions hold great promise in improving healthcare access, particularly in remote or underserved areas, and empowering patients to take an active role in managing their well-being.

Conventional pure-tone audiometry is conducted by presenting test tones in a specific frequency sequence, starting at 1,000 Hz and moving upward and then covering lower frequencies. Thresholds are determined by finding the lowest level at which responses occur. There are two methods for threshold measurements without masking: the ascending method, which identifies the lowest level in more than half of the ascents, and the descending method, which averages the lowest levels in both ascents and descents. If needed, masking noise is applied to the non-test ear during threshold measurements. The process of finding hearing thresholds has been algorithmically automated, allowing for testing to be conducted without the need for an examiner. Devices have been introduced to facilitate this automation, and further advancements have simplified the process to the extent that it can now be operated on smartphones^11–13^.

In this regard, audiometry with smartphone application has the benefits of being freely available to anyone, self-administered, and unrestricted by time or location. Performing pure tone audiometry in otolaryngology clinics requires trained professionals, hearing test equipment, and a sound-treated booth, all of which are expensive for patients. In many low-income countries, there aren't enough hearing health care workers, especially audiologists, speech pathologists, and ear, nose, and throat specialists^19^. Previous research has shown that utilizing application-based audiometry for the initial assessment of sudden sensorineural hearing loss or for screening hearing loss in elderly populations is both beneficial and reliable^11,13,20^. A meta-analysis highlighted the sensitivity and specificity of 89% and 93%, respectively, based on 25 studies with application-based audiometry^21^. This review emphasized the importance of patient's age, equipment, and the use of a soundproof booth, which were significantly associated with the diagnostic accuracy of application-based hearing tests^21^. In addition, a recent systematic review evaluated the accuracy of smartphone-based hearing screening tests in comparison to traditional audiometric tests^22^. Although it found high accuracy in application-based audiometry, the review noted that the quality of evidence was generally low^22^. It emphasized that further research is needed to ascertain the clinical utility and cost-effectiveness of these tests for mass screening purposes^22^.

The application used in this study employed earbud headphones that are easily accessible for everyone and was configured to operate when ambient noise levels were below 40 dB HL. Hearing thresholds were calculated in a manner similar to conventional audiometry. The study included a diverse range of patients with hearing impairments, and for individuals with normal hearing or mild hearing loss, the results were excellent and comparable to conventional audiometry.

The present study highlights the clear correlation between the outcomes of two examinations and the benefits of smartphone applications, it is important to acknowledge that there are still several limitations. It is plausible to speculate that the lower threshold results at high frequencies in the application-based audiometry might have derived from calibration errors, occlusion effects, or an increased response in the higher frequencies within the frequency response curve of the used earphones in this study. A previous review article analyzing applications for hearing test, also postulate the calibration process to achieve better performance, but it could not always be done for many application audiometry and additional evidence is required^23–25^. The discrepancy in hearing thresholds in the higher frequency range between application-based and conventional audiometry might be derived from the fact that non-audiometric headphones typically exhibit high sound pressure levels in the high-frequency area within the frequency response curve. In addition, this observation could be attributed to the testing environment, calibration issues, or occlusion effect^23^. Given the challenges associated with accurate calibration in app-based hearing tests, it may be necessary to provide detailed guidelines on the specifications for the devices and earphones used in such tests, as well as the testing environment, to ensure precise evaluations. This suggests that establishing clear standards for earphones and the testing environment is crucial for app-based hearing assessments.

In this study, the process of participants performing the app-based hearing test was monitored by the researchers, and data from tests that proceeded without issues were analyzed. However, as illustrated in Fig. 3, there were cases where the app-based hearing test results significantly differed from those of conventional audiometry. These discrepancies were primarily observed in older patients with asymmetric hearing loss. This indicates that factors such as the patient's hearing profile and age can influence the outcomes of app-based hearing assessments. Moreover, for subjects with asymmetric hearing, the present results showed inaccurate results with application-based audiometry in the worse hearing ear, which we assumed were derived from the presence of cross-hearing and the lack of a masking procedure. The absence of a masking process in the application may lead users to misinterpret their hearing levels, directly affecting the screening effectiveness of the application-based audiometry.

During this study, unforeseen issues came to light. It became evident that individuals with hearing loss, particularly elderly subjects, were not familiar with the use of smartphones or smart tablets, necessitating education to facilitate their effective utilization. Furthermore, we noted that the hearing assessment application used lacked a mechanism for users to pause or restart the test if they made mistakes or needed to interrupt the assessment, highlighting the need for sustained concentration during the testing period. Those limitations could potentially restrict the user base for app-based hearing assessments and specialized methods for hearing tests tailored to application-based audiometry, along with the development of user interfaces for the hearing-impaired, need to be established to increase the utilization of application-based hearing tests. In addition, enhancing features to address functional hearing loss and reliability of test results would increase the utility of theses assessments.

## Conclusion

The increasing prevalence of hearing loss underscores the growing importance of hearing screening. This study elucidated the hurdles and constraints associated with substituting conventional audiometry administered by trained audiologists with smartphone application-based audiometry. Nonetheless, the merits of smartphone-based audiometry, encompassing its broad accessibility, self-administration capability, and cost-effectiveness, in conjunction with the demonstrated correlation in this investigation, unequivocally establish the feasibility of employing smartphone-based audiometry as an effective hearing loss screening tool especially in symmetric hearing.

## Data Availability

The datasets used and/or analyzed during the current study available from the corresponding author on reasonable request.
